# Dry Needling and Antithrombotic Drugs

**DOI:** 10.1155/2022/1363477

**Published:** 2022-01-07

**Authors:** María Muñoz, Jan Dommerholt, Sara Pérez-Palomares, Pablo Herrero, Sandra Calvo

**Affiliations:** ^1^Universidad San Jorge, IPhysio Research Group, Zaragoza, Spain; ^2^Bethesda Physiocare, Myopain Seminars, Bethesda, MD, USA; ^3^Department of Physical Therapy and Rehabilitation Science, School of Medicine, University of Maryland, Baltimore, MD, USA; ^4^IIS Aragon, University of Zaragoza, Department of Physiatry and Nursing, Faculty of Health Sciences, Zaragoza, Spain

## Abstract

Many clinicians increasingly use dry needling in clinical practice. However, whether patients' intake of antithrombotic drugs should be considered as a contraindication for dry needling has not been investigated to date. As far as we know, there are no publications in analyzing the intake of antiplatelet or anticoagulant agents in the context of dry needling techniques. A thorough analysis of existing medications and how they may impact various needling approaches may contribute to improved evidence-informed clinical practice. The primary purpose of this paper is to review the current knowledge of antithrombotic therapy in the context of dry needling. In addition, reviewing guidelines of other needling approaches, such as electromyography, acupuncture, botulinum toxin infiltration, and neck ultrasound-guided fine-needle aspiration biopsy, may provide specific insights relevant for dry needling. Based on published data, taking antithrombotic medication should not be considered an absolute contraindication for dry needling techniques. As long as specific dry needling and individual risks are properly considered, it does not change the risk and safety profile of dry needling. Under specific circumstances, the use of ultrasound guidance is recommended when available.

## 1. Introduction

Worldwide, clinicians are using dry needling (DN) to reduce pain [1, 2], increase range of motion and flexibility [3], enhance performance [4], reduce spasticity [5], or improve fascial and scar tissue mobility [6, 7]. Dry needling is a safe and cost-effective treatment approach [8–11], but there is no literature informing clinicians whether DN can be applied safely in patients with altered coagulation. Potentially, patients taking antithrombotic drugs may have an increased risk of suffering bleeding complications when being treated with DN. The American Physical Therapy Association recommends that “patients with an abnormal bleeding tendency must be needled with caution. Dry needling of deep muscles that cannot be approached with direct pressure to create hemostasis may need to be avoided to prevent excessive bleeding” [12]. The most common target of DN is trigger points, which are located in close proximity to endplate zones [13]. Dry needling may also target fascia and fascial adhesions [6, 13, 14], but since veins and arteries are never the therapeutic targets, the risk of bleeding is significantly reduced. During DN, the needle may inadvertently penetrate blood vessels before reaching its therapeutic target, which may be relevant to patients taking antithrombotic agents. Some caution is warranted, especially when DN is targeting deeper muscles in close proximity of major arteries and veins, such as the maxillary artery when needling the lateral pterygoid muscle [15].

In an Irish study of the possible adverse events of DN, 39 physiotherapists used trigger point DN 20 times per month within a 9-month time frame for a total of 7,629 treatments. Mild adverse events, including bleeding, bruising, and pain during and after treatment, were reported in 19.18% of the treatments. While no significant adverse events occurred, the risk of significant adverse events for 10,000 treatments was estimated to be less than 0.04% [8]. Boyce et al., using a similar research design to Brady et al., reported 7,531 minor adverse events and 20 major adverse events out of a total of 20,494 DN treatments by 420 physiotherapists over a period of 6 weeks [10], which corresponded to 36.7% of the treatments resulting in a minor adverse event. The top three minor adverse events were bleeding (16%), bruising (7.7%), and pain during DN (5.9%) [10]. Comparing the two studies, it is not clear why the adverse event rate in Boyce et al.'s study was nearly twice as high as in Brady et al.'s study, but in both studies, bleeding was a common minor adverse event. There is no universally accepted system for reporting adverse events of DN, and therefore, the actual incidence of dry needling-related adverse events is unknown [16].

There are many specific factors to consider before using DN, such as the actual needling procedures, the area to be needled, and the characteristics of the needles. Specific factors to be considered are the diameter and type of needle, the technical procedure, the target of the intervention, and the anatomical structures in proximity of the needling site. Much can be learned from studies of electromyography (EMG), acupuncture, botulinum toxin (BTX) infiltration, and neck ultrasound-guided fine-needle aspiration biopsy (USGFNAB), realizing the obvious differences between these procedures and DN with respect to clinical practice or the type of needles used.

Dry needling is used mostly by physiotherapists, but other disciplines have also started to use DN, such as occupational therapy, athletic training, chiropractic, and acupuncture. Since these clinicians may not have a solid working knowledge of antithrombotic drugs and their specific risk factors, it is imperative to review the different kinds of antithrombotic drugs before reviewing guidelines from other procedures that use needles.

## 2. Antithrombotic Medications

There are two classes of antithrombotic drugs: antiplatelet agents and anticoagulants. The main antiplatelet agents are aspirin and P2Y12 receptor blockers. Anticoagulants include vitamin K antagonists (VKAs) and direct oral anticoagulants (DOACs), which are administered orally, and heparins, which are administered subcutaneously or intravenously.

Aspirin, the first-line antiplatelet, prevents the formation of blood clots and is used in patients with angina, previous heart attack or stroke, coronary artery stents, after coronary artery bypass graft surgery, and in patients undergoing surgery for hip fracture [17]. Other antiplatelet agents are the P2Y12 receptor blockers, such as clopidogrel, prasugrel, and ticagrelor. It is very frequently used as dual antiplatelet therapy (DAPT) with aspirin plus a P2Y12 receptor inhibitor to avoid thrombosis following balloon angioplasty or angioplasty with stent implantation. The main risk of DAPT is bleeding [18].

The main types of oral anticoagulant agents are VKAs and DOACs. Warfarin is the most widely used VKA worldwide, while in Europe acenocoumarol and phenprocoumon are the most frequently prescribed VKAs [19]. They are prescribed for the prevention and treatment of patients suffering from venous thromboembolism (VTE) or the prevention of systemic embolism or stroke in patients with prosthetic heart valves or atrial fibrillation (AF) [20]. In recent years, DOACs have been developed to overcome some of the limitations of VKAs, and they are expected to be used more widely in the coming years [20]. Currently, the US Food and Drug Administration (FDA) has approved five DOACs: dabigatran, rivaroxaban, apixaban, edoxaban, and betrixaban [21].

Finally, unfractionated heparins (UFH) and low molecular weight heparins (LMWH) have been used for decades. UFH are administered subcutaneously or intravenously. LMWH, derived from UFH, are administered subcutaneously and have gradually replaced UFH for most clinical indications. UFH and LMWH are prescribed, along with dual antiplatelet therapy in patients with acute coronary syndrome (ACS) and they reduce VTE complications of high-risk medical conditions, such as heart failure or prolonged immobilization in bed, or in knee or hip arthroplasty [22].  Table 1 summarizes the different types of antithrombotic drugs, their generic and brand names, their mechanism of action, and other specific information.

## 3. Bleeding Risks Related to Drugs

During VKA therapy, the risk of bleeding is assessed with the International Normalized Ratio (INR), which is a standardized method for reporting results of the prothrombin time (PT assay) [31]. Typically, an INR range of 2.0–3.0 is recognized as the therapeutic range and recommended by international guidelines [32]. Some reports assumed a lower risk of stroke with an INR range between 2.0 and 3.5 and an increased risk with INR values below 2.0 or above 3.5 [33–35]. An optimal time in the therapeutic range (TTR >75%) is associated with a lower risk of adverse events [36, 37]. VKAs are effective and widely used, but their use can be problematic because of several drawbacks (see Table 1). The risk of major bleeding is significantly lower in patients with a stable INR control. However, even optimal maintenance therapy within the therapeutic range does not necessarily avoid all possible bleeding complications. Many individual factors can influence the INR. Therefore, routine monitoring of the INR, appropriate clinical control, and continuous patient education are required [19].

DOACs have equivalent or improved therapeutic profiles compared to warfarin, and they are associated with significant reductions in stroke, intracranial haemorrhage, and mortality [30]. As the mechanism of action of DOACs differs from that of VKAs, the results of INR tests are unreliable in patients treated with these new agents [38]. Currently, there are several available tests to assess the anticoagulant effects of DOACs, but they have poor sensitivity and specificity, providing limited information [39]. DOACs do have predictable pharmacological profiles, which implies that they can be given in fixed doses without the need for routine therapeutic monitoring [29]. Despite their benefits, DOACs do have some drawbacks, such as certain drug-drug interactions [40].

Factors influencing blood clotting must also be considered (see Table 2). The FDA-approved reversal agent specific for VKAs is 4-Factor Prothrombin Complex Concentrate (4F-PCC) [50]. The FDA-approved antidotes readily available for DOACs are idarucizumab for dabigatran, and andexanet alfa for rivaroxaban and apixaban. However, Prothrombin Complex Concentrate (PCC) may be used off-label for reversing DOACs. Ciraparantag is a universal reversal agent, which is in development [39].

In case of major bleeding, it is recommended to withhold antithrombotic drugs and provide mechanical hemostasis and hemodynamic support [51]. Numerous studies have compared bleeding rates between different anticoagulant agents, as well as for different pathologies, but any comparison remains difficult due to different bleeding definitions, differences in individual trials, and various populations with different bleeding risks [52]. If there is a high thromboembolic risk, concomitant therapy is administered with an oral anticoagulant and an antiplatelet agent. It is more effective in reducing the risk of death and thromboembolism than a VKA alone, but it increases the risk of major bleeding, especially with long-term use, and in some cases may outweigh the benefits [25, 53, 54]. Concomitant therapy may also include the administration of aspirin and a P2Y12 blocker. Regarding the bleeding risk associated with antiplatelet agents, each agent needs to be considered individually due to different profiles (Table 2).

## 4. Other Factors Affecting Bleeding Risks

Moreover, other factors such as age [41], gender [42], interactions with food and drugs [40, 43, 44, 55], renal function [37, 41], and possibly exercise [56, 57] have to be considered. More specific details are shown in Table 2. Being 75 years or older is considered a risk factor in stroke risk-stratification schemes, as the prevalence of arterial and venous thromboembolic diseases and other risk factors, such as hypertension or diabetes mellitus, is higher in elderly adults [41]. Regarding gender, a review assessing the evidence of increased thromboembolic risk in women with AF suggests that the female gender is an independent stroke risk factor, but the causes of increased thromboembolic risk in women have not yet been fully determined [42].

In relation to the interaction between food and anticoagulants, Violi et al. concluded that the available evidence does not support current advice to restrict dietary vitamin K intake while taking VKAs. However, they recommended maintaining a stable diet avoiding wide variations in vitamin K [55].

Leite et al. analyzed the interferences of medicinal plants with blood hemostasis and warfarin anticoagulation and found a total of 58 different plants that may alter blood clotting and anticoagulation with warfarin [43]. Regarding drug interactions of VKAs, many medications influence their anticoagulant effect, such as anti-infectious and cardiovascular drugs, analgesics, anti-inflammatories, immunologic, central nervous system drugs, and gastrointestinal drugs. DOACs also interact with several drugs [40, 44]. In relation to renal insufficiency, Szummer et al. concluded that severe chronic kidney disease patients with an estimated glomerular filtration rate (eGFR) of less than 30 mL/min have a worsened INR control while taking warfarin. However, an optimal time in the therapeutic range (TTR >75%) is associated with a lower risk of adverse events, independently of underlying renal function [37]. When comparing DOACs with VKAs/warfarin, LMWH, aspirin, and placebo in patients with renal insufficiency, the recommended doses were noninferior and relatively safe compared with conventional anticoagulants, and all had a comparable efficiency [41].

Further research is needed to assess the influence of physical activity on the pharmacokinetics of antithrombotic drugs. Shendre et al. demonstrated that regular physical activity in patients on chronic anticoagulation therapy with warfarin is associated with higher dose requirements and a lower risk of haemorrhage [57], which was confirmed by Rouleau-Mailloux et al. [56].

## 5. Specific Risks of Other Needling Therapies and Interventions

Considering the type of needle, the degree of potential tissue damage is dependent on whether the needle is bevelled. The potential for tissue damage and bleeding is greater with a hypodermic needle than with a solid filament needle used with DN. Other needling approaches use different types of needles. For example, the needle diameter used with DN is smaller than the typical gauge of needles used for diagnostic EMG or hypodermic needles, ranging from 35 G to 28 G or 0.14 mm to 0.35 mm, respectively, compared to gauges of 30 G (0.30 mm), 26 G (0.45 mm), or 23 G (0.60 mm) with EMGs [58]. Interestingly, EMGs usually do not produce significant bleeding or hematomas [59]. Factors that may influence the patient's bleeding risk with these other needling procedures may be applied cautiously to the context of DN.

Another factor is the technical procedure itself. Typically, DN involves manipulating the needle within a muscle and fascia to elicit the so-called local twitch responses [1]. Theoretically, the risk of injury and bleeding may increase when multiple insertions are performed or when the needle is moved back and forth within the muscle. However, the small diameter and shape of the needle usually do not cause much concern. Of course, clinicians may opt to use needles with smaller diameters and limit the number of passes through a muscle. Another factor is the target of the intervention, which requires thorough anatomical knowledge of the location and the common distribution of blood vessels and nerves.

## 6. Electromyography

Several EMG studies have evaluated the incidence of hematoma and the risk of bleeding after the procedure in patients taking antithrombotic medication [59]. Two cases of symptomatic bleeding while performing an EMG were reported with patients who had abnormal blood coagulation and who were taking antithrombotic medication. Rosioreanu et al. reported a case of a pseudoaneurysm of the calf after an EMG in a patient with AF who was taking warfarin [60]. Butler et al. reported a case of a significant subcutaneous haemorrhage in a patient on chronic anticoagulant therapy, which required a two-unit blood transfusion [61]. Lynch et al. evaluated the risk of hematoma formation in patients taking antithrombotic medication compared to controls after standard needle EMG of the tibialis anterior muscle followed by ultrasound. There were no hematomas in the control group (51 patients). Of 101 patients taking warfarin, two had small, subclinical hematomas, and one out of 57 patients taking clopidogrel or aspirin had a small, subclinical hematoma. None of the patients had symptomatic bleeding. Therefore, there were no statistically significant differences between groups [62].

Gertken et al. established the incidence of Magnetic Resonance Imaging- (MRI-) detectable hematomas after EMG of the paraspinal muscles. The sample included patients taking antithrombotic agents and controls. A total of 431 spine segments and a total of 370 patients were reviewed with 139 patients taking aspirin, eight taking clopidogrel, ten taking warfarin, two taking heparin, and two taking LMWH. In this study, no paraspinal hematomas were observed in any group, which suggests that EMG of paraspinal muscles is a relatively safe procedure [63]. These results do vary from those obtained by London et al. in which possible hematomas were found in patients undergoing MRI after EMG. In this study, the risk of paraspinal hematoma formation was assessed by MRI scans after performing extensive EMG of the lumbar paraspinal muscles using the paraspinal mapping technique, which involves inserting the needle at multiple levels on both sides of the back. Of 29 patients who underwent MRI after EMG, six had possible hematomas, but they were not clinically relevant, and there were no significant bleeding complications [64]. Lee and Kushlaf assessed the risk of bleeding after needle EMG in patients taking DOACs. A 19-item survey questionnaire was sent to 3,959 members of the American Association of Neuromuscular and Electrodiagnostic Medicine (AANEM) and 58 responded. Fifty-four (93%) responders performed needle EMG on patients taking DOACs, 13 (22%) responders had written laboratory guidelines specific to perform EMG on patients taking anticoagulants, and 4 (7%) responders had written laboratory guidelines including DOACs. Seven (12%) responders asked patients to withhold DOACs before performing EMG. One responder mentioned that the period for withholding DOACs depended on the medication. Two (3%) responders had known patients with thrombotic complications when discontinuing DOACs. A single (2%) responder reported a case of a small asymptomatic hematoma in the paraspinal muscle region detected by post-EMG MRI [65].

## 7. Acupuncture

Lee et al. found that increasing the diameter of acupuncture needles significantly increased the incidence of bleeding-related adverse events in patients taking anticoagulants or antiplatelet therapy, especially more in the head, face, and feet regions [66]. A prospective adverse effects acupuncture study by Witt et al. showed that the most common adverse effects were bleedings or hematoma [67]. McCulloch et al. reported that acupuncture appears safe in patients taking anticoagulants citing a 0.003% complication rate, assuming adequate needling location and depth. Bleeding events with acupuncture were limited to asymptomatic bruises or minor drops of blood after removing the needle. The only serious reported bleeding events were due to inappropriately deep needling (acute carpal tunnel syndrome induced by haemorrhage and cecal intramural hematoma in a patient taking anticoagulant agent) or by aggressive concomitant anticoagulant therapy itself (intracranial haemorrhage) [68].

A retrospective chart review of 242 patients and 4,891 acupuncture treatments concluded that acupuncture appeared safe for patients taking warfarin or antiplatelet medications [69]. The World Health Organization (WHO) published a systematic review of the Chinese-language literature on acupuncture-related adverse events based on 115 articles (98 case reports and 17 case series). One of the most common acupuncture-related adverse events was subarachnoid haemorrhage, which was reported in 35 patients of the 296 cases [70]. As with DN, there is no universally accepted system for reporting adverse events of acupuncture [16, 71]. Lee et al. evaluated the risk of bleeding-related adverse events (microbleeding, hematoma, and ecchymosis) after acupuncture treatment in patients taking anticoagulant or antiplatelet therapy; 169 patients taking anticoagulants or antiplatelet therapy (exposure group) and 259 patients taking no medication (nonexposure group) were assessed immediately after acupuncture treatment and before acupuncture treatment the following day. Mild bleeding-related adverse events occurred in 38.5% of patients in the exposure group and 44.4% of patients in the nonexposure group. Microbleeding was most common in both groups (70.0% and 59.1% in the exposure and nonexposure group, respectively). Results showed that the use of such medication did not increase the incidence of bleeding-related adverse events in acupuncture treatment, and most of the adverse events related to bleeding were mild [66]. Kwon et al.'s study also demonstrated that acupuncture is safe in patients taking DOACs. Recorded medical data about bleeding-related side effects immediately after needle removal in patients taking DOACs versus those patients taking antiplatelet agents and no anticoagulant therapy were retrospectively reviewed. One hundred-sixteen patients received 10,177 acupuncture sessions during a 9-month period, and the incidence of microbleeding was 3.9% in the DOAC group compared with 5.6% and 5.1% in the antiplatelet and the control group, respectively [72].

## 8. Botulinum Toxin Injections

Studies of botulinum toxin (BTX) injections and potential bleeding complications must be considered with some caution as the infiltrations are generally made with larger and bevelled-edged needles compared to the smaller solid filament needles used with DN. Nevertheless, a review of possible BTX bleeding complications may help as injections are commonly used for the management of spasticity in patients taking antithrombotic agents. Only a few studies have attempted to determine the rates of bleeding complications, the particular physicians' practice of performing the injections, and common preferences to control the risk of bleeding prior to the injections. For example, a Korean survey of physiatrists found a high variability between physicians with respect to injecting patients on anticoagulant therapy, and generally, there was a tendency to avoid BTX injections all together. Seventy percent of the respondents considered the INR value prior to performing the injections in patients taking anticoagulants. The majority of respondents thought that INR values between 2 and 3 were optimal. However, many others replied that an INR value lower than 2 was the preferred range. Most respondents used an INR value measured 2–7 days prior to the injection and acknowledged that they did not have access to standardized prevention protocols for performing BTX injections. Thirty-one percent of the respondents used some preventive measures, such as applying prolonged compression at the injection site and observing signs of swelling or bruising after the injection [73].

In a 2012 study by Schrader et al., 20 patients on the oral anticoagulant phenprocoumon with optimal INR values received BTX therapy using a 27 G (0.40 mm) needle without producing an increase in hematoma formation [74]. In relation to the previous study and after a follow-up period of four years, Schrader et al. published an article in 2017 concluding that none of the hematomas were surgically relevant and that the risk of minor hematoma after BTX therapy in patients taking anticoagulants is slight and only occurs in periocular injections [75]. Phadke et al. conducted a retrospective review of medical charts of patients with spasticity who received BTX injections with 26 G (0.45 mm) needles in their leg compartment muscles. Patients were taking anticoagulants, antiplatelet medications, or no medication. INR values were below 2.5. The authors found no evidence of compartment syndrome or bleeding events in the superficial or deep compartment locations [76].

In summary, there are no established and validated criteria, recommendations, or consensus for BTX injections for patients taking anticoagulants [73, 77]. With an INR ≤2.6 and 27 G (0.40 mm) or thinner needles, BTX injections do not pose a risk in patients [74, 76].

## 9. Ultrasound-Guided Fine-Needle Aspiration Biopsy

USGFNAB is the most accurate technique to evaluate thyroid nodules, and many patients who are candidates for this procedure are on anticoagulant treatment [78]. The needle used in this technique is a thin, fine-gauge needle, usually 25 G (0.50 mm) or 27 G (0.40 mm), which is smaller in diameter than typical hypodermic needles, but bigger than the needles used for DN. A study of USGFNAB bleeding risks concluded that the aspiration did not increase the formation of hematomas, nor did it pose an increased risk of major bleeding. Discontinuing anticoagulant therapy with VKAs before the procedure was not necessary. There are no specific published studies of patients taking DOACs who undergo USGFNAB but continuing the medication prior to this procedure is safe [78].

## 10. Recommendations

There are no studies comparing the incidence and prevalence of adverse events associated with DN in subjects taking antithrombotics and subjects who do not take these medications. It is also not known whether some of the relatively mild adverse effects in general populations may become more severe for patients taking anticoagulants.

Clinicians must always be cautious when applying DN techniques, considering the use of medications that affect coagulation. Besides, it is necessary to have other competencies, especially excellent anatomical knowledge. It is mandatory to complete a thorough clinical evaluation, which should include an inspection of the skin prior to DN looking for signs of excessive bruising [59]. According to the “Analysis of Competencies for Dry Needling by Physical Therapists,” published by the Federation of State Boards of Physical Therapy in the US, of the 116 entry-level and 22 dry needling-specific knowledge requirements, 117 were identified as important for competency in DN [79].

Although not directly related to DN, some professional associations have recommended specific considerations that may be considered for DN. For example, AANEM has advised that an EMG evaluation should start with small superficial muscles before proceeding to deeper ones, although according to Boon et al., standard needle EMG of potentially high-risk muscles in patients taking antithrombotic drugs does not imply an increased risk of hematoma formation [58, 80]. Currently, as in EMG procedures, there is no evidence to support postposing DN routinely because of antithrombotics use, and medications should not be discontinued before the procedure [59]. Other examples are the recommendations of the AANEM [80] and the WHO guidelines on drawing blood [81]. In patients who are at higher risk of bleeding, the AANEM recommends to control hemostasis throughout the procedure by applying prolonged direct compression after removing the needle over the insertion site to minimize the risk of bleeding and bruising [80]. The WHO Guidelines recommend using a gauge of 19–23 and a length of 1–1.5 inches (except for a 19–20 gauge for which this is not applicable) for drawing blood in adults. After withdrawing the needle and syringe, firm and sustained pressure must be applied to stop any bleeding for as much as 2–3 minutes or 5 minutes or more for patients on anticoagulants. Moreover, to reduce the risk of extensive bleeding the WHO also recommends using a needle gauge smaller than the actual vein [81]. Considering drawing blood versus DN, it is important to consider that with DN techniques, veins are not purposely punctured. Besides, needles used with DN do not have a cutting bevelled edge like those used for blood draws or BTX infiltration, so they will cause less tissue damage. Moreover, the small diameter of the needles minimizes the likelihood of bleeding [15].

In conclusion, with DN, the pressure is usually maintained for about 5 seconds for nonanticoagulated patients; for patients taking anticoagulants the pressure should be applied for about 10–15 seconds. Despite the low risk of bleeding and bruising with DN in patients who are taking medications that affect coagulation, it is important to be cautious when needling close to major blood vessels, or when applying hemostasis is not an option. The patient's individual risks must be considered as previously described, similar to EMGs and other needling procedures [59].

The use of sonography in Doppler mode is recommended, but not essential, with DN of particular muscles such as the tibialis posterior, lateral pterygoid, or psoas major muscle, which not only reduces the chance of penetration major vessels but also increases the accuracy of the technique. Although sonography is increasingly used in clinical practice, most clinicians do not have access to sonography.

## 11. Conclusions

Clinicians must always inform patients about the possible risks of DN and obtain at least an oral informed consent prior to DN. When specific information about the patient's coagulation status, such as the INR, is not readily available, clinicians should determine the potential risk factors by including specific questions during the clinical interview, such as the patient's experience with bleeding following venipuncture. When a given patient presents with diminished coagulation, clinicians may consider initially reducing the intensity of the DN techniques. Although the bleeding response for every patient may vary between days or even on the same day, it is recommended to observe the patient's bleeding response and initially avoid DN in deeper muscles until its safety has been established with more superficial muscles, especially with patients on anticoagulant therapy. Applying prolonged hemostasis following DN is recommended.

Venipuncture and other invasive procedures, such as USGFNAB and botulinum injections, are performed regularly in patients who are taking anticoagulants, and they have been proven to be safe. They do not cause a major bleeding event. Therefore, taking antithrombotic medication should not be considered an absolute contraindication for DN techniques. If specific dry needling and individual risks are properly considered, antithrombotic medications do not change the risk and safety profile of dry needling. For improving the safety of DN practice in these patients, the use of ultrasound guidance is recommended, but not essential, for DN in locations close to major blood vessels or in deeper muscles where hemostasis cannot be applied.

## Figures and Tables

**Table 1:** Summary of antithrombotic drugs characteristics.

Type of drug		Generic names	Brand names^*∗*^	Observations	Mechanism of action
Antiplatelets	Nonselective inhibitor of COX [23]	Aspirin	*Ascriptin,* *Ecotrin, Ecpirin*	Causes gastrointestinal irritation and bleeding that can be reduced by the intake of a proton pump inhibitor (e.g., omeprazol) [17]	Nonselective inhibitor of COX [23]
	P2Y12 receptor blockers	Clopidogrel	*Plavix*	High interindividual variability, delayed onset of action, and bleeding episodes in certain cases [17]	Irreversible inhibitors of P2Y12 receptors in platelets [24, 25]
		Prasugrel	*Effient*	Faster onset of action, less interindividual variability but higher risk of bleeding vs. clopidogrel [25, 26]	
		Ticagrelor	*Brilinta*	More benefits vs. clopidogrel and prasugrel [27]	Reversible inhibitor of P2Y12 receptors [27]

Anticoagulants	VKAs	Warfarin	*Coumadin, Jantoven*	Used for decades for patients that need long-term oral anticoagulation [28] Slow onset of action, narrow therapeutic window, genetically determined inter- and intrapatient variability, and multiple possible interactions with various foods and drugs [19]	Indirect mechanism of action by inhibiting the synthesis of coagulation factors [29]
		Acenocoumarol	*Sinthrome, Sintrom*		
		Phenprocoumon	*Falithrom,* *Marcoumar*		
	DOACs	Rivaroxaban	*Xarelto*	Faster onset and offset of action vs. VKAs [28], lower bleeding risk vs. warfarin in younger patients but not in those >75 years [30] Much more expensive vs. VKAs, but lower resource consumption vs. cost of VKAs and the required therapeutic monitoring [19] Betrixaban is not indicated for treatment. It is used as a prophylactic agent for prevention of deep vein thrombosis and pulmonary embolism in adults hospitalized for an acute medical illness [21]	Direct inhibition of factor Xa [28]
		Apixaban	*Eliquis*		
		Dabigatran	*Pradaxa, Pradax*		
		Edoxaban	*Savaysa, Lixiana*		
		Betrixaban	*Bevyxxa*		

Abbreviations: COX: cyclooxygenase enzyme; VKAs: vitamin K antagonists; DOACs: direct oral anticoagulants. ^*∗*^There are many other brand names for some of the medications.

**Table 2:** Specific details of factors affecting bleeding.

Factor	How it affects
Age	Conventional risk factors, comorbidities, and malignant disease in elderly adults increase the risk of bleeding and VTE [41]. In younger patients, DOACs were associated with lower bleeding risk compared with warfarin but there were no statistically significant differences in >75 years [30].

Gender	Factors proposed to explain the increased thromboembolic risk in women: increased hypertension, renal dysfunction, hyperthyroidism, increased hypercoagulability, cardiovascular remodeling, and estrogen hormone replacement therapy, as well as specific gender influences on the quality of the anticoagulant treatment (i.e., lower quality of warfarin anticoagulation in females with AF, which requires higher rates of anticoagulation prescription that increases the risk of bleeding [42].

Food interactions	Warfarin is affected by a wide range of targets in blood hemostasis, including inhibition of COX, the presence of coumarins and other substances, or high amounts of vitamin K. Herbs with the greatest potential to interact with warfarin include ginseng, garlic, ginkgo, St. John's wort, and ginger, but even menthol cough drops may reduce the INR [43].

Drug-drug interactions	Some examples of medication affecting VKAs: (a) Drugs, including ciprofloxacin, cotrimoxazole, cephalosporins, fluconazole, and metronidazole, can increase the warfarin effect. (b) Several cardiovascular drugs can potentiate the metabolism of warfarin and increase the INR, including aspirin, amiodarone, antihyperlipidemic agents, and statins, such as fluvastatin, lovastatin, and simvastatin. (c) Analgesics, including phenybutazone, piroxicam, acetaminophen, and NSAIDs, can increase the anticoagulation effects. (d) Central nervous system drugs, such as antidepressants, citalopram, fluoxetine, paroxetine, and tricyclics antidepressant can increase the INR and the risk of bleeding. (e) Alcohol is a risk factor with concomitant liver disease. (f) Gastrointestinal drugs, such as cimetidine and omeprazole, can increase the INR [44]. Some examples of medication affecting DOACs: (a) Dabigatran interacts with antacids, which decrease the effect of dabigratan; (b) Antiarrhythmic agents, such as amiodarone, verapamil, quinidine, as well as antiplatelet agents, LMWH, and nonsteroidal anti-inflammatory drugs (NSAIDs), increase the anticoagulant effects [40]. (c) Rivaroxaban interacts with antiacids, antifungical medications, such as itraconazole, voriconazole, and posaconazole, antiplatelet agents, NSAIDs, such as naproxen, and LMWH among others increasing the anticoagulant effects [40].

Renal impairments	In mild renal insufficiency (eGFR: 50–79 mL/min), the major bleeding risk was lower with any DOACs than with warfarin. In moderate renal insufficiency (eGFR: 30–49 mL/min), the risk was higher, with rates of major bleeding of 6.8% versus 4.8% in patients with mild insufficiency and a trend toward less major bleeding with the DOACs [41].

Exercise	A study carried out on three patients taking warfarin showed an inverse relationship with increased physical activity and decreased INR. Thus, it may be possible that an increase in physical activity puts patients at greater risk of thromboembolism [45–47]. Ryan et al.'s study showed that taking aspirin before running 60 minutes increased both the intestinal and gastroduodenal permeability of aspirin compared with taking a placebo and running or placebo plus rest but not aspirin plus rest. Nevertheless, the clinical significance of this study is highly questionable [48]. In a study by Sawrymowicz et al., 20 healthy patients took an oral dose of aspirin 1 g and then walked on a treadmill at 4.8 km/h for 20 minutes per half hour for 3 hours. Blood samples taken during the exercise did not show changes in plasma concentration, clearance, or half-life compared with a 3-hour rest [49].

Abbreviations: COX: cyclooxygenase enzyme; DOACs: direct oral anticoagulants.

## Supplementary Materials

Supplementary Materials
*Supplementary File 1.* Dry needling of the abductor digiti minimus muscle.
