# Supplementary material for: Dry Needling and Antithrombotic Drugs
Source: Pain Res Manag. 2022 Jan 7;2022:1363477. doi: 10.1155/2022/1363477 (PMC8759918; doi:10.1155/2022/1363477)
Supplement: Supplementary Materials — Supplementary File 1. Dry needling of the abductor digiti minimus muscle. [file 1363477.f1.docx]

Please visit the below link to view the requested video of Abductor Digiti Minimus Hand.mp4:

https://drive.google.com/file/d/1XAdhdnTsa2q70I26bTdMosWGRHEUS2rm/view
